# Inequalities in Exposure to Ambient Air Neurotoxicants and Disparities in Markers of Neurodevelopment in Children by Maternal Nativity Status

**DOI:** 10.3390/ijerph18147512

**Published:** 2021-07-14

**Authors:** Faven Araya, Jeanette A. Stingone, Luz Claudio

**Affiliations:** Department of Environmental Medicine and Public Health, Icahn School of Medicine at Mount Sinai, New York, NY 10029, USA; faven.araya2@mssm.edu (F.A.); js5406@cumc.columbia.edu (J.A.S.)

**Keywords:** air pollution, neurotoxins, neurodevelopment, nativity, maternal, disparities, child development

## Abstract

Exposure levels to environmental pollutants vary significantly among different populations. These inequities in exposure to hazardous air pollutants (HAP) among different populations can contribute to disparities in neurodevelopmental outcomes. The aim of this study was to determine if exposure to HAP varies by maternal nativity status, a demographic marker often overlooked in the study of health disparities. We also assessed if those inequalities in exposure levels are associated with neurodevelopmental measures in young children. To do this, we obtained data from the Early Childhood Longitudinal Study-Birth cohort (ECLS-B), a nationally representative sample of children born in the U.S. in the year 2001 (*n* = 4750). Bayley’s Short Form-Research Edition (BSF-R) was used to measure cognitive development at 2 years of age. Using residential location at nine months of age, participants were assigned exposures to ten HAPs identified as potentially neurotoxic. Linear regression models were used to assess the joint effect of maternal nativity status and HAP exposure on neurodevelopment. Results showed inequities in exposure levels to ten different HAPs among the populations, as approximately 32% of children of foreign-born mothers were exposed to high levels of HAPs, compared to 21% of children born to U.S.-born mothers. Adjusting for socioeconomic factors, both isophorone exposure (a marker of industrial pollution) (−0.04, 95% CI, −0.12, 0.04) and maternal nativity status (−0.17, 95% CI, −0.27, −0.06) were independently associated with lower standardized BSF-R mental scores in children. Interaction between nativity status and isophorone was not statistically significant, but the change in mental scores associated with isophorone exposure was greater in children of foreign-born mothers compared to children of U.S.-born mothers (−0.12, vs. −0.03, *p* = 0.2). In conclusion, exposure to HAPs within the highest quartile was more commonly found among children of foreign-born mothers as compared to children of US-born mothers, indicating inequities in pollutant exposure by nativity status within urban populations. Exposures associated with nativity status may negatively contribute to children’s neurodevelopment.

## 1. Introduction

Disparities in neurodevelopment have been described in children with different socio-demographic indicators [1,2,3]. Children in communities of color, immigrant and low-income families have been shown to be at high risk for adverse health and developmental outcomes [1,2,3,4,5,6]. Immigrant health is of particular interest due to the increasing influx of global migration, including within the United States (US). Conflicting theories regarding immigrant health challenges what is understood about the relationships between socioeconomic indicators and health outcomes [7,8]. With the immigrant population projected to double by 2050, understanding the determinants of immigrant health and their children’s health is essential in understanding how future health outcomes will be shaped in the US [9]. 

One way in which health disparities arise between populations of different socioeconomic status is through differences in exposure levels to environmental toxicants [2,10]. In particular, children’s neurodevelopment is highly sensitive to exposure to air pollutants [11,12]. For example, growing evidence has shown that exposure to hazardous air pollution is concentrated in urban areas, disproportionately affecting minorities and low-income communities, and may therefore contribute to the observed differences in neurodevelopment [13,14,15,16]. Elevated pollutant concentrations are highly prevalent in urban areas, largely due to motor vehicle traffic, industrial activities and biomass burning [17].

Exposure to hazardous air pollutants (HAP), defined by the US Environmental Protection Agency (EPA) as “toxic air pollutants harmful to human health”, has been linked to adverse neurodevelopmental outcomes such as autism and attention deficit disorder (ADD) [18,19,20,21,22,23,24,25]. In addition, many HAPS commonly found in urban environments have been identified as being disruptive to early brain development, including benzene, diesel, ethylbenzene, chloroform, toluene, styrene, manganese, polycyclic aromatic hydrocarbons (PAH)/polycyclic organic matter (POM), xylene, and isophorone [18,26,27,28,29,30,31,32,33]. Growing evidence highlights prenatal exposures to HAPs contributing to the disruption of fetal brain development introducing vulnerabilities to adverse neurodevelopment in children [31,34,35]. Previous studies showed the relationship between early life exposure to benzene, toluene, ethylbenzene, and xylene (BTEX), and neurodevelopment, as measured by the development of attention deficit/hyperactivity disorder (ADHD) as well as greater use of academic support services in children highly exposed to this form of air pollution [36]. Similarly, other studies have shown that early life exposure to diesel and PAH/POM, additional markers of pollution due to vehicular traffic, were associated with lower cognitive development scores in children [29,37,38]. Other pollutants such as chloroform, styrene, manganese and isophorone, which are typically emitted by urban stationary industries, are known neurotoxins and are also found to be associated with cognitive developmental deficits [26,28]. Previous studies have found early life and chronic exposure to these industrially-emitted pollutants are associated with lower cognitive development scores [39], autism [40], and brain tumors [41,42]. A recent study found racial and ethnic disparities in school-based environmental exposures to neurotoxicants and proposed the potential negative impact on school-based performance [43]. Though the exact mechanism by which these air pollutants affect neurodevelopment is unknown, their neurotoxic properties suggest that they have the potential to contribute to neurodevelopmental deficits in children and may contribute to disparities among different populations [19,44]. 

Neurodevelopmental disparities among populations are complex and have multifactorial outcomes. In a previous study, the interaction between exposures to air pollutants and the social home environment on neurodevelopmental outcomes was investigated and found them to independently influence standardized math test scores in urban children [45]. A recent study using national-level datasets examined children’s residential exposures to vehicular HAPs, and found racial and ethnic disparities also noting disproportionately high exposures among children from non-English speaking homes, an indicator of nativity status [46]. Using a similar approach, the objective of this study is to further the investigation of disparities in neurodevelopmental outcomes in children by assessing HAP exposure profiles by maternal nativity status. We hypothesize that maternal nativity status is an important, but often overlooked factor when examining neurodevelopmental disparities caused or aggravated by exposure differences to air pollution during childhood. 

## 2. Methods

This study was reviewed and approved by the Institute of Education Sciences Data Security Office and by the Institutional Review Board of the Icahn School of Medicine at Mount Sinai.

### 2.1. Study Population

We obtained data from the Early Childhood Longitudinal Study Birth ECLS-B cohort, a nationally representative sample of approximately 10,700 children born in the U.S. in 2001. All sample sizes throughout this study were rounded to the nearest 50 per the data requirements of the National Center of Education Statistics (NCES). Data on maternal demographics, children’s residential ZIP code, and children’s neurodevelopmental assessments from the ECLS-B were collected and used for analysis. Children were enrolled at 9 months and then followed through kindergarten entry, participating in interposed study visits designed to assess their cognitive, socio-emotional, and physical development [47]. The children’s assessments were supported by interviews and questionnaires administered to parents, child care professionals, and education providers detailing the characteristics and experiences that may be associated with their development and kindergarten readiness [47]. Using these data, comparisons were made to identify neurodevelopmental differences among children of U.S.-born and foreign-born mothers exposed to hazardous air pollutants. Maternal nativity was used due to 95% of primary caregivers identifying as biological mothers for the children [48]. Eligibility was limited to those children whose data on pollutant exposure was available and who had completed the cognitive assessments at the age 2 study visit. Exclusions were made for those who did not reside in urban areas and did not have complete information on influential variables (*n* = 4750). Restriction to urban residence was done to appropriately capture the populations at the greatest risk of exposure to high levels of hazardous air pollution and to control for socioeconomic indicators that may vary by urbanicity of residence and confound associations between environmental exposure and neurodevelopment. Maternal nativity status was assigned based on self-reported birth country and categorized as either born in the U.S. or foreign-born. All children were US born, but for the purpose of this study were assigned nativity status based on maternal nativity. 

### 2.2. Outcome Assessment 

Obtained from ECLS-B cohort, measures of neurodevelopment were assessed using the Bayley’s Short Form-Research Edition (BSF-R), an adaptation of the Bayley Scales of Infant Development-Second Edition (BSID-II) [47]. This modified assessment tool was used to measure a child’s babbling, vocabulary, active exploration, understanding of repetitive actions, and problem solving skills near their nine month and two year milestones [47]. The BSF-R mental scores obtained from the assessment administered at the study visit when children were approximately two years old and were used to represent neurodevelopmental outcomes in this study. Distribution of scores did not meet normality assumptions, and were therefore standardized using a mean of zero and a standard deviation of one [49]. Thus, these BSF-R mental z-scores were used in all further analyses as the measure of children’s neurodevelopment. 

### 2.3. Exposure Assessment

For this study, we prioritized ten hazardous air pollutants previously identified as suspected neurotoxins in humans and present in the urban environment [26]. Selected pollutants included benzene, diesel, ethylbenzene, chloroform, toluene, styrene, manganese, PAH/POM, xylene, and isophorone. Obtained from the 2002 National Air Toxics Assessment (NATA) database, pollutant exposure profiles in the population were based on modeled annual ambient concentration estimates and used for analysis. NATA serves as the U.S. census-tract level air toxic evaluation tool for the Environmental Protection Agency (EPA). We assigned individual exposure levels by linking the NATA database to the children’s residential zip code at nine months. NATA estimates ambient concentrations of pollutants at each census-tract in the US. In order to obtain exposure estimates for a child’s zip code of residence, we constructed weighted average exposures for each zip code using the percent of residential housing within each zip code that lies within each census-tract. These data were obtained from the Office of Housing and Urban Development and United States Postal Service ZIP Crosswalk files. This method is consistent with previous research linking NATA to ECLS-B data [50]. Rural-Urban Continuum Codes linked to each child’s zip codes were used to determine urban residence. 

We compared pollutant exposure profiles among children of foreign-born and U.S.-born mothers. Individual and collective exposure measures were evaluated, but only isophorone displayed a crude association with BSF-R mental z-scores and was subsequently used for further assessments. To account for the rightly-skewed distribution, isophorone along with the other nine pollutants were dichotomized at the 75th quartile to obtain ‘high’ and ‘low’ exposure categories [32,36]. This approach reduces exposure measure susceptibility to overly influential outliers occurring when totaling the individual-level estimated exposure concentrations [32]. Estimated isophorone exposures greater than the 75th quartiles (≥7.90 ng/m^3^) were categorized as ‘highest quartile’ exposure and exposures below the 75th quartile (<7.90 ng/m^3^) were deemed as ‘lowest quartile’ exposures. Similarly, a composite variable summing all ten individually dichotomized pollutants was created. This variable was designed to represent a marker for children exposed to the highest quartile across all ten pollutants. Highest and lowest quartile exposure categories for all pollutants were relative to our study parameters and did not reflect or exceed EPA standards. 

### 2.4. Identification of Influential Variables

Influential variables that may have affected the outcome of interest and exposures were identified through directed acyclic graph analysis and review of existing literature. Selected variables included: maternal race, maternal education, birth weight, socioeconomic status (SES), and neighborhood deprivation index (NDI) [20,36,51,52]. We used four classifications to identify race/ethnicity: White (non-Hispanic), Black (non-Hispanic), Hispanic, and Asian (non-Hispanic). Other classifications were collapsed and combined to account for low frequency counts in other racial/ethnic classes. We also collapsed maternal education attainment into three broad categories: those who received a high school education and below, those who received some college education, and those who received a bachelor’s degree or above. Maternal education was limited to highlight demographic characteristics of the study population and was not controlled for in the analysis due to it being captured by the SES variable. We classified birth weights obtained from birth certificates as: normal birth weight (weighing >2500 g at birth), low birth weight (1500 ≥ 2499 g at birth), and very low birth weight (weighing <1500 g at birth). ECLS-B constructed a quintile-based SES variable that incorporated parental education, occupational prestige, and household income [47]. The NDI was calculated using each child’s census-tract residential zip code in conjunction with several census-based socio-demographic factors identified through principal component analysis [53]. Retained from the analysis, the following variables from the 2000 U.S. census were used to construct the NDI: percent of males in management and professional occupations, percent of crowded housing, percent of female-headed households with dependents, percent of households on public assistance, percent below the federal poverty line, percent earning less than a high school education, and the percent unemployed [53]. A greater NDI corresponds to a greater amount of deprivation and lower level of neighborhood SES. 

### 2.5. Statistical Analysis

We conducted descriptive analyses using chi square to assess the demographic characteristics of the population based on exposure status. Survey design accommodated for complex sampling methods including weighting, stratification, and clustering. Using linear regression models, we assessed independent associations between each of the two exposure parameters and neurodevelopment. Multiple linear regression models were used to assess the joint effects of maternal nativity status and exposure to HAPs on BSF-R mental z-scores. Crude and adjusted effect estimates were reported with corresponding confidence intervals. Effect modification was explored through the inclusion of an interaction term between maternal nativity status and isophorone exposure. All associations were assessed at alpha level 0.05. All analyses were performed using SAS version 9.4 (SAS, Cary, NC, US).

## 3. Results

### 3.1. Diversity in the Demographic Characteristics of the Population

**Figure 1:**
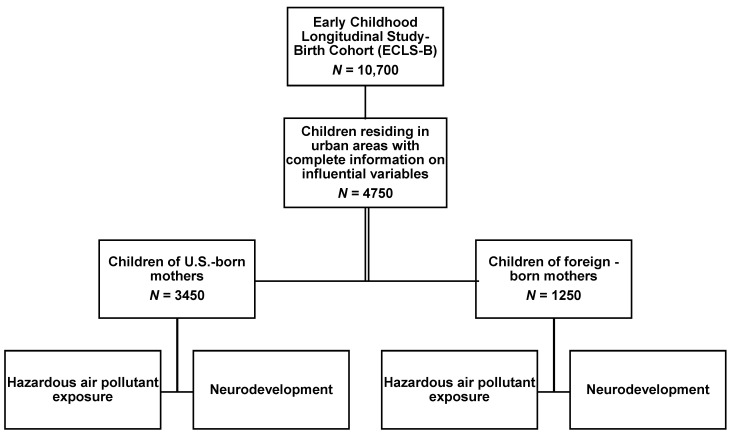
Flow-diagram illustrating the selection of study population from the ECLS-B (2001 birth cohort). All sample sizes are rounded to the nearest 50 per data requirements of NCES.

**Figure 2:**
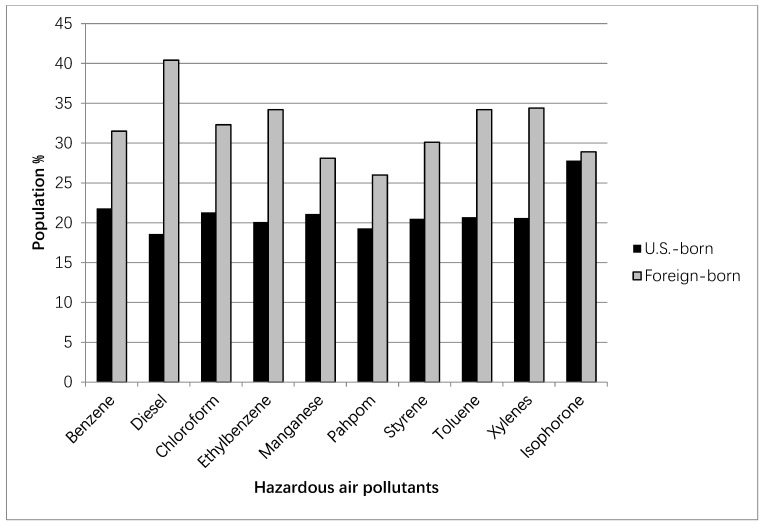
Highest quartile exposure to hazardous air pollutants by maternal nativity status in the ECLS-B population, by nativity status (2001 birth cohort).

The total population was comprised of 4750 children, of which 3450 (74%) were children of U.S-born mothers and 1250 (26%) were children of foreign-born mothers (Figure 1). Demographic characteristics of the sample population are described in Table 1. Children of U.S-born mothers were more likely to be born to mothers who were White, non-Hispanic, have some college education, and are more likely to fall within the lowest socioeconomic quintile. Children of foreign-born mothers were more likely to be Asian, non-Hispanic, have a mother with a bachelor’s degree or higher, and live in a household likely to fall within the highest socioeconomic quintile. Among children of U.S-born mothers, 950 (28%) were exposed to levels of isophorone ≥7.90 ng/m^3^ (Figure 2). Similarly, children of foreign-born mothers, 350 (29%) were also exposed to levels of isophorone ≥7.90 ng/m^3^.

### 3.2. Assessing Air Pollutant Exposure profiles

We compared pollutant exposure profiles among children of U.S-born mothers and those of foreign-born mothers (Figure 2). Cumulative exposures within the highest quartile of benzene, diesel, chloroform, ethylbenzene, toluene, styrene, manganese, PAH/POM, xylenes, and isophorone were highest among children of foreign-born mothers across all ten pollutants. On average, we found approximately 32% of children of foreign-born mothers were exposed to the highest quartile of HAP compared to 21% of children of U.S.-born mothers.

### 3.3. Maternal Nativity Status Associated with Measures of Cognitive Development

We examined the relationship between maternal nativity status and cognitive development in the children. No differences were observed for models including the composite variable, therefore individual level results were used. Crude and adjusted effect estimates of nativity status on neurodevelopment are presented in Table 2. Results of crude effect estimates show BSF-R mental z-scores of children of foreign-born mothers to be 0.37 [95% CI: −0.46, −0.28] lower than children of U.S.-born mothers. After adjusting for maternal race, birth weight, socioeconomic status, and neighborhood deprivation index, BSF-R mental z-score effect estimates were 0.17 [95% CI: −0.29, −0.06] lower among children of foreign-born mothers when compared to children of U.S.-born mothers.

### 3.4. Air Pollutant Exposure Assessment

We assessed each pollutant individually to determine its relationship with neurodevelopment in children. Only isophorone exposure showed an association with neurodevelopment and was therefore used for further analysis. Crude and adjusted effect estimates of isophorone exposure within the highest quartile on neurodevelopment are presented in Table 2. In crude models, isophorone exposure within the highest quartile was associated with a lower BSF-R mental z-score of −0.14 [95% CI: −0.24, −0.04]. After adjusting for sociodemographic factors and NDI, the relationship between isophorone exposure within the highest quartile and BSF-R mental z-scores was slightly attenuated −0.05 [95% CI: −0.12, −0.004]. 

Inclusion of both maternal nativity status and pollutant exposure within adjusted models was performed to assess the independent effects of maternal nativity and pollutant exposure on BSF-R mental z-scores (Table 2). Models that included both isophorone (−0.04, 95% CI, −0.12, 0.034) and maternal nativity status (−0.17, 95% CI, −0.29, −0.07) continued to show independent associations with lower BSF-R mental z-scores, as estimates barely changed from the single-exposure models. Children of foreign-born mothers continued to have lower BSF-R mental z-scores, suggesting that additional environmental and/or social factors that were not accounted for in our study may explain the disparities in neurodevelopment observed among children of foreign-born mothers. 

### 3.5. Interaction between Isophorone Exposure, Maternal Nativity on Neurodevelopment

We introduced an interaction term into the model to assess the joint effect of maternal nativity status and isophorone exposure on neurodevelopment. Interaction between nativity status and isophorone was not statistically significant, but the change in BSF-R mental z-scores associated with isophorone exposure was greater in children of foreign-born mothers compared to children of U.S.-born mothers (−0.12 95% CI −0.23, −0.01, vs. −0.03 95% CI −0.10, 0.03, *p*-value on interaction term = 0.18). 

## 4. Discussion

The role of maternal nativity status on neurodevelopmental differences was previously described in the context of socio-demographic factors, but more information about how environmental exposures may influence health in these populations was lacking [6,49,54]. Findings from that study highlight disproportionately high exposures to vehicular HAPs among children from non-English speaking households. Similarly, in this study, we observed elevated exposure to all ten neurotoxic air pollutants we assessed in children of foreign-born mothers, indicating inequities in pollutant exposure by nativity status within urban populations. Isophorone exposure, a marker of industrial air pollution, was the only pollutant significantly associated with negative effects on neurodevelopment, indicated by lower BSF-R mental z-scores. The independent effect of isophorone exposure on neurodevelopment was persistent even when controlling for maternal nativity. Despite adjusting for isophorone exposure, deficits in BSF-R mental z-scores among children of foreign-born mothers persisted, even though foreign-born mothers were not low in SES indicating the presence of additional contributing factors. Interaction between maternal nativity and pollutant exposure was not statistically significant, but a reduction in BSF-R mental z-score associated with isophorone exposure was greatest [55] among children of foreign-born mothers. This suggests that the effect of isophorone exposure on neurodevelopment may be greater among children of foreign-born mothers than for those with mothers born in the US. 

Our findings related to neurodevelopment and maternal nativity are not consistent with sociodemographic indicators of health outcomes used to characterize immigrants [3]. While immigrant communities are often described as low-income, less educated, and more vulnerable to poor health outcomes, our findings highlight lower mental z-scores among foreign-born mothers despite being more educated and having higher SES than their US counterparts. This contributes to the highly varied literature of the theorized immigrant paradox [55]. In our study, maternal foreign-born status did not serve as a protective characteristic for their U.S.-born children as proposed by the immigrant paradox, nor did the more desirable sociodemographic determinants of health. Assessment of environmental exposures was used to address the role it may play in the deficits we observed due to its link to adverse neurodevelopmental outcomes previously established in the literature [20,21,26,27,56,57]. Lower BSF-R mental z-scores associated with isophorone exposure within the highest quartile may serve as an indicator that early-life chronic environmental exposures can lead to adverse neurodevelopmental effects. This has been exhibited in previous studies where severe air pollution exposure was shown to be associated with neuro-inflammation and structural brain alterations resulting in child cognitive deficits [27,30,58]. In addition, a recent review of studies over a ten year period assessing the relationship between air pollution and cognitive functions in children showed that isophorone was linked to lower math skills [28]. While isophorone exposure categories used for this study are relative and do not reflect or exceed EPA standards, early and low-dose chronic exposure may affect neurodevelopmental outcomes.

Densely populated industrialized cities are known for poor air quality due to high emission rates of HAPs caused by industrialization [16,59]. Our study population was restricted to urban areas in order to capture those at the greatest risk of exposure to these kinds of pollutants. In addition, studies have provided evidence that residential environments of specific communities are disproportionately exposed to localized physical and chemical toxicants that contribute to persistent health disparities [43,60]. Neighborhoods in which low income, minority or immigrant populations reside tend to have higher levels of many pollutants [15,61,62,63]. Evaluation of exposure profiles by maternal nativity status exhibited inequities in the distribution of exposure to hazardous air pollutants within the highest quartile among children of foreign-born mothers across all ten pollutants. Sources of environmental contaminants within these residential environments were not evaluated within this study, but differential land-use and industrial activity may be contributing factors to the observed differences in exposure profiles. Differences in exposure profiles and subsequent BSF-R mental z-scores among nativity groups alludes to the impact of environmental exposures, but the results of our independent assessment of both exposures suggest the potential for additional influences. 

There are some limitations to our study. Pollutant exposures were based on 2002 NATA annual ambient estimated mean pollutant concentrations. These exposures were linked to a child’s address at nine months because address at birth was not available, limiting our ability to make associations with early exposures to neurotoxicants. This estimated measure of annual pollutant concentration compromises the ability to draw temporal associations and introduces susceptibility to misclassification bias [64]. Pollutants have been previously determined to be highly correlated with one another, and presumably correlated with other pollutants not assessed in this study [40]. Misclassification and collinearity may lead to the misrepresentation of the true effect estimates of independent exposures and need to be considered when assessing significant associations [64]. Also, selection of isophorone for further assessment was due to the lack of significant associations among the remaining nine pollutants, but it is unclear if isophorone is itself causally related to neurodevelopmental effects or if it is a marker for exposure to industrial activities, although some other evidence suggests that it can cause measurable neurofunctional changes in children [28]. Other forms of non-differential misclassification were possible as this study lacked information on individual exposure assessment. Also, our stratum specific populations may not have been sufficiently powered to detect an interaction, though a slight change in BSF-R mental z-score was observed in children. Small stratum specific sample populations also prevented specific information on nativity country and/or region of origin and maternal length of residence in the US to be identified and evaluated. Lastly, limitations include the lack of information available to assess pollutant exposures among foreign-born mothers prior to arriving in the US. Maternal age at US arrival for foreign-born mothers was not explicitly provided, but data highlighted over 65% migrated as adults. Sociodemographic characteristics and exposure profiles may vary by maternal length of time in the US and need to be further assessed in future studies. 

In spite of these potential limitations, this paper examines an important factor in children’s environmental health: the potential effects of exposure to air pollution to children’s brain development [12,65,66]. We believe that this paper contributes to our understanding of nativity-related environmental health disparities. 

## 5. Conclusions 

Exposure to hazardous air pollutants within the highest quartile was greatest among children of foreign-born mothers. Higher exposure levels to one particular air pollutant, isophorone, among children of foreign-born mothers showed an association with deficits in neurodevelopment. However, this association did not completely explain the disparities in BSF-R mental z-scores at age 2 that were observed among children of foreign-born mothers. Further exploration of the role of nativity status is needed to better understand its potential impact on health outcomes. 

## Figures and Tables

**Table 1:** Demographic characteristics in the ECLS-B study population and stratified by maternal nativity status (2001 birth cohort).

Maternal Nativity Status			
Demographic Factor	Total Population (*n* = 4750) (%)	U.S.-Born (*n* = 3450) (%)	Foreign-Born (*n* = 1250) (%)
Maternal Race			
White, non-Hispanic	47.20	45.05	2.16
Black, non-Hispanic	17.88	16.28	1.59
Hispanic	18.23	9.12	9.12
Asian, non-Hispanic	16.19	1.68	14.51
Other, non-Hispanic	0.49	0.34	0.16
Maternal Education			
High school and below	39.20	39.20	39.00
Some college	27.80	30.70	19.60
Bachelor’s Degree & above	33.00	30.10	41.40
Birth weight			
Normal	75.20	71.34	86.60
Moderately low	15.20	17.44	8.42
Very low	9.54	11.21	5.02
SES Index Quintile			
First	14.80	13.88	17.22
Second	17.40	17.74	16.33
Third	19.60	21.44	14.39
Fourth	19.80	21.75	14.63
Fifth	28.30	25.19	37.43
NDI mean (SD)	−0.11 (1.02)	−0.11 (1.00)	−0.12 (1.09)

**Table 2:** Crude and adjusted effect estimates and 95% confidence intervals estimating the effect of maternal nativity status and isophorone exposure on mental z-scores in the ECLS-B population (2001 birth cohort).

Characteristic	Model 1	Model 2	Model 3 ^a^	Model 4 ^a^	Model 5 ^a^
	Estimate [95% CI]	Estimate [95% CI]	Estimate [95% CI]	Estimate [95% CI]	Estimate [95% CI]
Nativity status					
U.S-born	*reference*		*reference*		*reference*
Foreign-born	−0.37 [−0.46, −0.28]		−0.17 [−0.29, −0.06]		−0.17 [−0.27, −0.06]
*Isophorone*					
≤7.90 ng/m^3^		*reference*		*reference*	*reference*
≥7.90 ng/m^3^		−0.14 [−0.24, −0.04]		−0.05 [−0.13, 0.004]	−0.04 [−0.12, 0.04]

^a^ Adjusted for maternal race, birth weight, socioeconomic status, and neighborhood deprivation index.

## Data Availability

No new data were created or analyzed in this study. Data sharing is not applicable to this article.
